# Notice of redundant publication: Can the difference in medical fees for self and donor freeze-thaw embryo transfer cycle, be in fact a cover-up for the sale of donated human embryos?

**DOI:** 10.1186/1747-5341-2-15

**Published:** 2007-08-03

**Authors:** Anonymous

Please note that a commentary recently published in this journal [1] includes substantial duplication of Letters to the Editor published in *Developing World Bioethics *[2] and *Human Fertility *[3].

The letter in *Developing World Bioethics *[2] was first published online on 6th March 2006 and included in the April print edition; this article [2] has since been retracted. The commentary in this journal [1] was published on 29^th ^March 2007. The letter in *Human Fertility *[3] was published in June 2007.

The author, Dr Heng, would like to apologise to the Editors and readers of *Philosophy*, *Ethics and Humanities in Medicine*, for failing to indicate the similarity of the article in this journal [1] to the others whilst it was under submission, and for the resulting redundant publications.

It is the journal and publisher's policy for notices of redundant publication to be authored by the author of the original article [2]. On this occasion, the original author refused.
